# Placental Chorangiocarcinoma: Case Report with Literature Review of a Rare Entity

**DOI:** 10.5146/tjpath.2021.01548

**Published:** 2022-09-15

**Authors:** Nishant Sagar, Parul Tanwar, Nita Khurana, Poonam Kashyap

**Affiliations:** Department of Pathology, Maulana Azad Medical College, New Delhi, India; Department of Obstetrics and Gynaecology, Maulana Azad Medical College, New Delhi, India

**Keywords:** Chorangiocarcinoma, Chorangioma, Trophoblastic proliferation, Placenta

## Abstract

Chorangiocarcinoma is an extremely rare tumor seen in the placenta, with only six cases reported in the literature so far. Its morphological characteristics, criteria for diagnosis, and the pathophysiology remain controversial to date. Although it was predominantly considered a benign entity, a solitary case of distant metastasis has been reported in the literature.

We present a case of this unusual tumor in the preterm placenta of a 29-year-old female. Grossly seen as a grey white nodule, microscopic examination revealed nests of atypical trophoblastic proliferation surrounded by vascularized stroma. No evidence of basement membrane invasion was noted. On immunohistochemistry, the trophoblastic component expressed pancytokeratin, Beta HCG, and Placental Alkaline Phosphatase with high Ki-67 labelling index.

The present case highlights this exceedingly rare entity with emphasis on its morpho-immunohistochemical features along with a review of literature.

## INTRODUCTION

Chorangiocarcinoma is an unusual malignant trophoblastic tumor of the placenta with only six cases reported in literature so far (1–6). Jauniaux et al. first described chorangiocarcinoma as a lesion showing a combination of both vascular and trophoblastic proliferation (1). Placental chorangiomas, on the other hand, are purely vascular benign lesions, similar to hemangiomas elsewhere in the body (7). The pathophysiology of chorangiocarcinoma is still unclear, although multiple hypotheses have been proposed. Due to a paucity of cases, its prognosis and management protocol also remain uncertain. We, herein, describe the seventh case of this entity with a review of the literature.

## CASE REPORT

A twenty-nine-year-old female presented to the emergency room at 30 weeks of gestation, with complaints of vaginal discharge and fever for one day. The patient was G4P1L1A2, and had a known history of moderate anemia (Hb 9 g/dl) and hypothyroidism. A clinical diagnosis of chorioamnionitis with premature rupture of membrane was made. Ultrasonography (USG) revealed a small hypoechoic lesion in the right lower abdomen in relation to the uterine fundus with maintained uterine contour. No other significant changes were observed on USG. The patient underwent caesarian section and delivered a preterm baby. The placenta was sent for histopathological examination with a clinical suspicion of chorioamnionitis.

**Figure 1:**
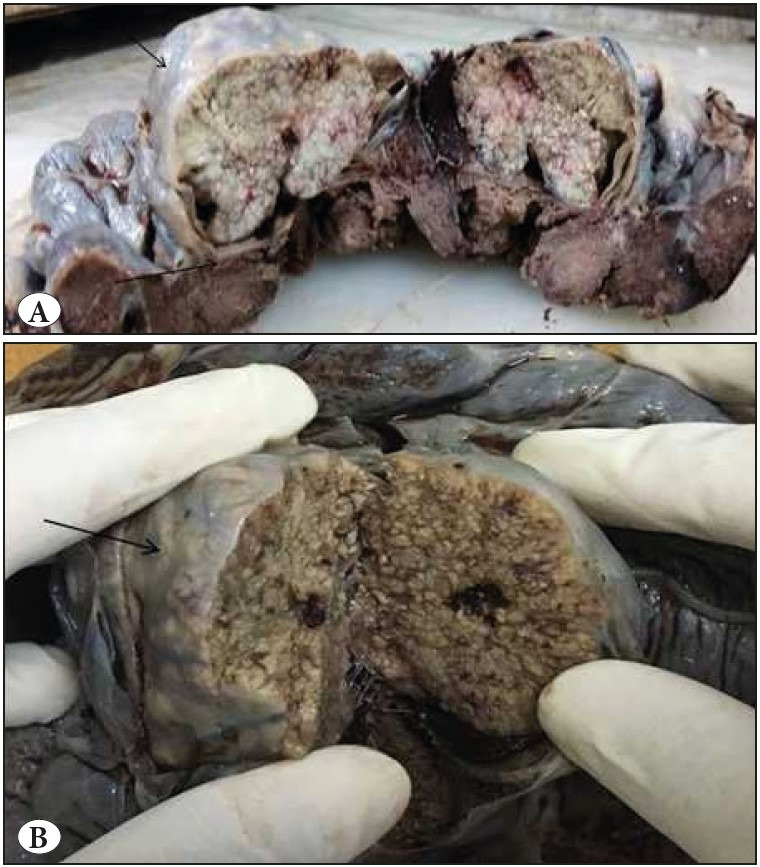
**A)** Well-circumscribed grey-white solid nodule seen in the placenta just below the membrane with no infiltration in the surrounding membranes or placental tissue. **B)** Cut section of the nodule showing friable and necrotic areas.

**Figure 2:**
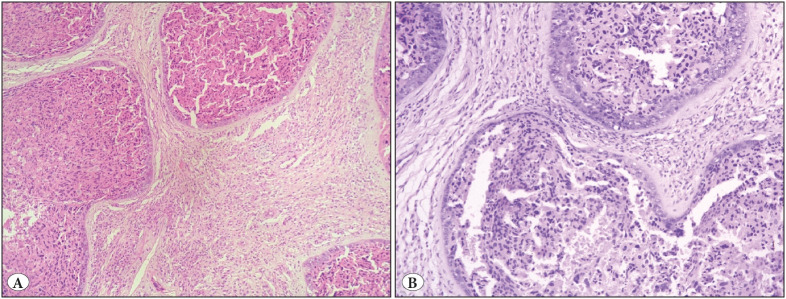
Multiple well-circumscribed nodules in a vascular stroma with central necrosis. No evidence of infiltration into the stroma. **A)** Low magnification (H&E, 100x), **B)** High magnification (H&E,400x).

**Figure 3:**
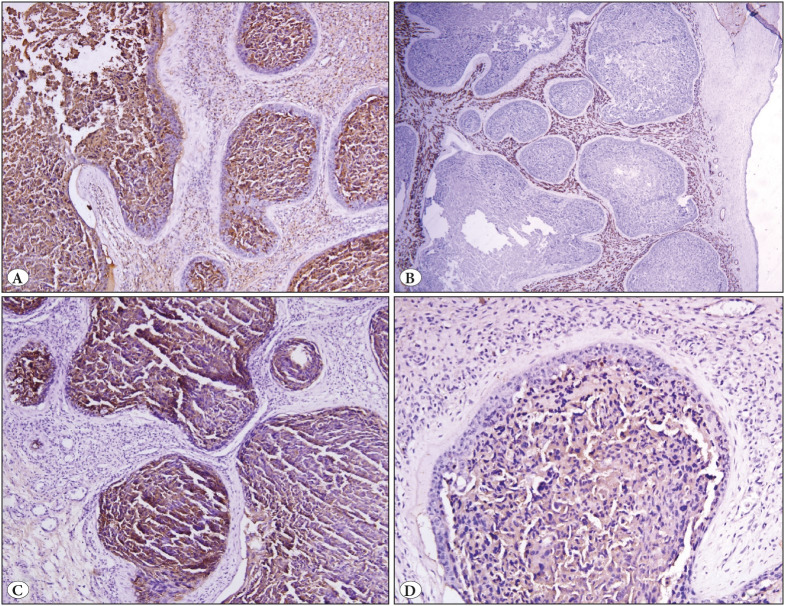
**A)** Bright beta HCG positivity in the central cells of the nodule (Beta HCG antibody, 100x). **B)** Stromal blood vessels highlighted by strong expression of CD34 (CD34 antibody, 100x). **C)** Strong expression of pancytokeratin by the tumor nests (PanCK antibody, 200x). **D)** Nests showed weak positivity for PLAP (PLAP antibody, 200x).

The placenta specimen measured 13x11x2 cm with the attached cord measuring 35 cm in length. Near the umbilical cord insertion, a grey white nodule was present measuring 5.5x4.5x3 cm. On gross examination, the nodule was well circumscribed showing no evidence of infiltration in the surrounding membranes or placental tissue. The cut section was solid-cystic with the presence of friable areas (Figure 1). Microscopic examination of the nodule showed multiple well-circumscribed large cellular nests dispersed in a vascular stroma. The nests were variably sized revealing a central area of moderately pleomorphic cells surrounded by cuboidal cells at the periphery. These cells in the center were polygonal in shape having a dense hyperchromatic nucleus, irregular nuclear borders, and a moderate amount of dense eosinophilic cytoplasm. Mitotic figures (5-6/10hpf), apoptotic bodies, and multinucleation was readily appreciated in these central cells. The peripheral cuboidal cells showed minimal pleomorphism. Areas of necrosis were identified at the center of the nests. The intervening stroma between the nests consisted of a large number of closely packed capillary sized vascular channels, lined by a single layer of endothelial cells, devoid of any significant pleomorphism or atypia (Figure 2). On immunohistochemistry (IHC), the nests exhibited strong expression of cytokeratin (AE1/AE3 clone, PathnSitu, USA) (Figure 3), Beta HCG (SPM105 clone, Thermo Scientific, USA) (Figure 3), Placental alkaline phosphate (PLAP) (PL8-F6 clone, BioGenex,USA) (Figure 3), and a high Ki-67 (GM001 clone, PathnSitu, USA) labelling index (90%) but no expression of p16 (JC2 clone, DBS Pleasonton, USA) was noted. CD34 (Qbend 10 clone, PathnSitu, USA) highlighted the endothelial lining of the capillaries in the intervening stroma (Figure 3). Surrounding placenta exhibited mild chorioamnionitis. Based on the presence of nodular proliferation of pleomorphic trophoblastic cells with a high proliferation index within a vascular stroma, a final diagnosis of chorangiocarcinoma was rendered.

## DISCUSSION

Chorangiocarcinoma was described, for the first time, by Jauniaux et al. in 1988 as a tumor having features of both chorangioma and choriocarcinoma. The authors categorized it as a malignant gestational trophoblastic tumor (1). Later, in 1994, Trask et al. described the second case having similar features as the first case, both being non-invasive (2).

All previously described cases of chorangiocarcinoma were found incidentally in term or near-term pregnancies. On macroscopic examination the tumor is seen within the placenta and grossly resembles placental infarct (1–6). Microscopic features of chorangiocarcinoma are not uniformly defined. The index case, described by Jauniaux et al, in 1988, showed villous vascular proliferation, surrounded by trophoblastic cells exhibiting pleomorphism and increased proliferative activity, similar to true trophoblasts (1). However, nineteen years later, in 2009, Ariel et al. described chorangiocarcinoma as having nodules of atypical trophoblastic proliferation within a typical chorangioma. The trophoblastic component exhibited frequent mitosis and necrosis (4).

Till date only six cases of chorangiocarcinoma have been published in the literature (Table 1). However, out of these, only three cases had features similar to the present case where the trophoblastic proliferation is found within the vascular stroma rather than surrounding it (4–6).

**Table 1:** Review of all the published cases of chorangiocarcinoma to date (1–6).

**Case No.**	**Author and year**	**Age** **(yrs)**	**POG** **(wks)**	**Clinical presentation**	**Maternal HCG levels**	**Morphological features**	**Necrosis**
**1 **	Jauniaux et al, 1988	35	35	C/S, vaginal bleeding	WNL	Chorangioma surrounded by atypical trophoblasts	**Absent**
**2**	Trask et al, 1994	36	36	NVD, twin	WNL	Chorangioma surrounded by atypical trophoblasts	**Absent**
**3**	Guschman et al, 2003	31	34	Fetal distress, IUGR	WNL	Chorangioma surrounded by atypical trophoblasts	**Absent**
**4**	Ariel et al, 2009	23	37	C/S, perineal condyloma	WNL	Nests of atypical trophoblasts within chorangiomatous stroma	**Present**
**5**	Faes et al, 2012	36	term	At term, normal	WNL	Nests of atypical trophoblasts within chorangiomatous stroma	**Present**
**6**	Huang et al, 2015	27	39	C/S	↑↑(F/U)	Nests of atypical trophoblasts within chorangiomatous stroma	**Present**
**7**	Present case, 2018	29	30	PROM, cord prolapse	WNL	Nests of atypical trophoblasts within chorangiomatous stroma	**Present**

**POG:** Period of gestation, **HCG:** Human chorionic gonadotropin, **C/S:** Caesarean section, **IUGR:** Intrauterine growth retardation, **WNL:** Within normal limits, **F/U:** Follow up, **PROM:** Premature rupture of membranes.

Lastly some groups suggested calling these tumors “chorangiomas with trophoblastic proliferations” and argued that the number of cases might be underrepresented in the literature. Khong presented a study on 33 placental chorangiomas and found “chorangioma with trophoblastic proliferation” in 15 out of 23 chorangiomas (65%) (8). According to Khong’s definition, all of these cases fulfilled the criteria for a chorangiocarcinoma diagnosis but the author avoided this terminology as there was no evidence of stromal invasion. Similarly, Ogino and Redline studied 70 chorangiomas, 50% of which had associated mild to moderate trophoblastic proliferation (7).

Except for one case, all previously described cases had a benign course with no evidence of metastasis at the time of delivery or during follow up (1–5). However, the last published case of chorangiocarcinoma by Huang et al. metastasized to the lung after three months of follow up post-delivery, indicating its malignant nature and justifying the term “chorangiocarcinoma” (6). Among the 7 cases discussed herein, including our case, 3 cases had the additional morphological feature of necrosis (Table 1), which is a high-grade feature beyond just trophoblastic proliferation. Interestingly, all of these three cases had nests of atypical trophoblastic cells within a chorangiomatous stroma, including the case with recurrence. Therefore, there might be two morphologic subgroups of these tumors, and cases with this particular morphology could be followed more carefully clinically.

The etiopathogenesis of chorangiocarcinoma remains uncertain. Upregulation of vascular growth factors have been hypothesized as a causative factor. However, Gurchman et al. did not find any significant difference in the expression of angiogenic factors (vascular endothelial growth factor, basic fibroblast growth factor, Ang-1, Ang-2 and Platelet-derived growth factor) in the lesion as compared with normal villi (3). Some authors have hypothesized that trophoblastic proliferation surrounding chorangioma may be due to the effect of certain angiogenic factors, but more studies are needed for confirmation (7).

The differential diagnosis of chorangiocarcinoma includes intraplacental choriocarcinoma and metastatic cervical squamous cell carcinoma. Vascular and stromal invasion are frequently described in intraplacental choriocarcinoma while it is absent in chorangiocarcinoma. Chorangiomatous vascular proliferation is seen in chorangiocarcinoma and not in intraplacental choriocarcinoma. However, the possibility of a chorangioma associated with a choriocarcinoma in situ cannot be entirely excluded as discussed previously (2,4). Cervical squamous cell carcinoma is positive for p16 and negative for PLAP (9).

Due to the paucity of cases, the appropriate management guidelines have not been described. The only described metastasizing case was administered etoposide-methotrexate-actinomysinD /etoposide-cisplatin chemotherapy and responded well (6). The present case showed no signs of metastasis for 6 months, after which she was lost in the follow up.

In conclusion, chorangiocarcinoma is a rare entity with a handful of cases reported in the literature. Although localized and non-infiltrative, these cases can metastasize, warranting close follow up. Differentiation of chorangiocarcinoma from the more common intraplacental choriocarcinoma is imperative to avoid an unnecessary aggressive treatment course.

## Conflict of Interest

The authors declare no conflicts of interest.
